# A 3D multi-agent-based model for lumen morphogenesis: the role of the biophysical properties of the extracellular matrix

**DOI:** 10.1007/s00366-022-01654-1

**Published:** 2022-05-06

**Authors:** Daniel Camacho-Gómez, José Manuel García-Aznar, María José Gómez-Benito

**Affiliations:** Department of Mechanical Engineering, Multiscale in Mechanical and Biological Engineering (M2BE), Aragon Institute of Engineering Research (I3A), University of Zaragoza, Zaragoza, Spain

**Keywords:** Agent-based model, Lumen morphogenesis, Fluid secretion, Mechanobiology, Computer simulations, Matrix density.

## Abstract

The correct function of many organs depends on proper lumen morphogenesis, which requires the orchestration of both biological and mechanical aspects. However, how these factors coordinate is not yet fully understood. Here, we focus on the development of a mechanistic model for computationally simulating lumen morphogenesis. In particular, we consider the hydrostatic pressure generated by the cells’ fluid secretion as the driving force and the density of the extracellular matrix as regulators of the process. For this purpose, we develop a 3D agent-based-model for lumen morphogenesis that includes cells’ fluid secretion and the density of the extracellular matrix. Moreover, this computer-based model considers the variation in the biological behavior of cells in response to the mechanical forces that they sense. Then, we study the formation of the lumen under different-mechanical scenarios and conclude that an increase in the matrix density reduces the lumen volume and hinders lumen morphogenesis. Finally, we show that the model successfully predicts normal lumen morphogenesis when the matrix density is physiological and aberrant multilumen formation when the matrix density is excessive.

**Supplementary Information:**

The online version contains supplementary material available at 10.1007/s00366-022-01654-1.

## Introduction

Lumen morphogenesis is a key process in the development of tissues and organs. Luminal structures are found in many parts of metazoan organisms, and they allow these organisms to perform specific functions, such as blood flow, digestion, and renal system activity. The formation involves coordination of cells in a sophisticated manner. Three basic principles are required to create a lumen: spatially controlled cellular mitosis, cell-cell and cell-matrix interaction, and cell fluid pumping [11]. However, how these mechanisms coordinate is not fully understood.

Fluid-solid interaction is critical in vascular biology [2], in hemodynamics [30], in angiogenesis [51] and, in general, in physiology and pathophysiology [19]. Thus, luminal structures are in a state of hydrostatic pressure [29], and this pressure, which is developed from fluid secretion, might be the driving force in luminal structures during morphogenesis [33], as shown in ductal network formation in the pancreas [9]. Furthermore, the mechanical properties of the extracellular matrix (ECM) play an important role in lumen formation. The mechanical interaction of cells with the ECM provides physical cues that are relevant to cellular self-organization [8]. An increment in tissue stiffness is related to tumorous behavior of cells, as tumors are generally stiffer than normal tissues [42]. In the case of luminal structures, matrix stiffening compromises tissue organization, inhibits lumen formation, and enhances growth [35]. This perturbation in the tissue architecture as a result of matrix stiffening produces a dysfunctional lumen associated with disease and may be related to the aberrant structures found in carcinomas [1, 12]. Therefore, understanding how these two factors influence lumen morphogenesis might provide insight into not only normal formation, but also pathological formation.

Previous computer-based models have been developed to study different aspects of luminal structures. Specifically, on-lattice models are the most prevalent. In this type of model, cells occupy sites in a regular lattice, and mechanical interactions are usually represented by minimizing the energy of the system [48]. Thus, [7] analyzed dynamic regimes of epithelial growth, [16] developed a predictive model that matches their in vitro data, [4] investigated two alternative mechanisms of lumen formation, [50] studied cell mechanics in embryonic bile duct, [39] simulated the development of an acinar structure with five different subpopulations of cells, and then the maintenance and stability of the epithelial monolayer and the hollow lumen [40]. Continuum approaches have been also adopted to simulate developmental processes. In the case of lumen morphogenesis, [10] studied the balance between paracellular leaks and the build-up of osmotic pressure in the lumen, and [14] built a continuum model to explore the role of the coupling of mechanical, electrical, and hydraulic phenomena in tissue lumen formation. Nonetheless, in this type of model, individual cells are neglected in favor of a larger scale of simulation, so it is difficult to take into account how heterogeneities in cell behaviors affect lumen initiation and formation from individual cells [45].

Although important achievements have been made, to the best of our knowledge, some crucial aspects have not yet been considered in lumen morphogenesis. Cell-cell interaction is essential and neither on-lattice models nor continuum models can directly represent the mechanical interaction between individual cells. Moreover, the forces produced by the luminal hydrostatic fluid pressure are essential in lumen morphogenesis, as they permit the lumen to initiate and expand. Finally, the mechanical properties of the ECM is usually neglected, even though it regulates the process, as it opposes the expansion produced by hydrostatic pressure. Therefore, we introduce a 3D multi-agent-based model for lumen morphogenesis that integrates the cell-cell and cell-matrix interactions, the hydrostatic pressure generation, and the role of the ECM. Agent-based models have been widely used to study cell and tumor growth [13, 20, 38], or tissue regeneration mimicking cell deformation [49]. Here, we aim to create a model that mimics lumen morphogenesis, taking into account the internal luminal hydrostatic pressure generated by cells’ secretion and the effect of matrix density. To evaluate the predictive capacity of the model, we qualitatively replicate the experimental results achieved by [35], who found that an increase in matrix stiffness inhibits lumen formation and enhances cell colony size, resulting in an aberrant multiluminal architecture.

## Materials and methods

**Fig. 1:**
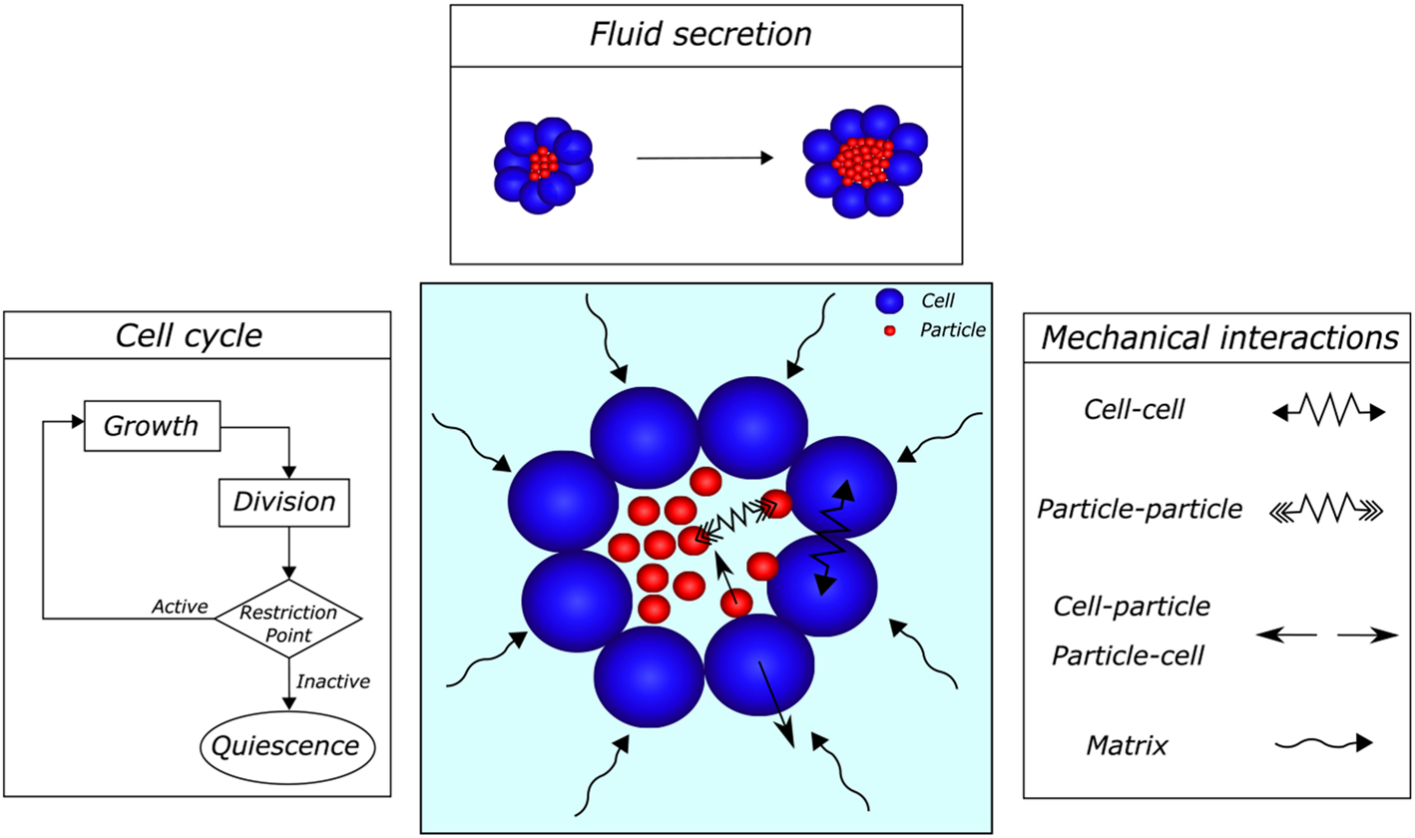
Sketch of the computational model, which consists of two main agents and their interactions. First, for the agent that simulates the cells, we consider a mathematical model for the cell cycle, which handles proliferation. Second, the fluid secretion agent makes cells secrete fluid into the lumen through particles, increasing the hydrostatic pressure. Finally, both agents interact mechanically, and the lumen expands

We formulate a three-dimensional multi-agent-based model for lumen morphogenesis, in which agents are spheres that do not change shape (Fig. 1). The objective of this computer-based model is to mimic the morphogenesis of an organoid composed of cells enclosing a fluid-filled lumen by means of numerical simulations. To accomplish this task, we consider two types of agents: cells, which are the biological entities, and particles, which are secreted by cells and simulate the lumen fluid.

Therefore, we define a computational model for simulating the cell cycle that regulates cell proliferation and fluid secretion to form the lumen. To simulate how cells secrete fluid, we assume that cells generate particles inside the lumen, thereby increasing the lumen volume. Consequently, the lumen is in a state of hydrostatic pressure [29] due to this cell secretion [18, 33].

Mechanical equilibrium between cells anchored to the ECM and the luminal pressure ensure the maintenance of the luminal architecture. To model this mechanical equilibrium, we use agents that interact mechanically to generate and maintain the lumen. Thus, cells interact among themselves based on pairwise potential functions in an adhesive-repulsive manner. Moreover, particles also interact among themselves via pairwise potential functions and interact with cells in a repulsive manner. This interaction mimics the luminal hydrostatic pressure generated by cells’ fluid secretion, and it is responsible for the movement of cells and generation of the luminal space. Finally, agents interact with the extracellular matrix by means of a friction coefficient that represents the dynamic viscosity of the matrix.

### Modeling the cell cycle

The cell cycle is a complex process that occurs that involves the growth and proliferation of cells, organismal development, regulation of DNA damage repair, tissue hyperplasia in response to injury, and diseases such as cancer [43]. Overall, it can be described by a growth phase, in which the cell progressively increases its volume as a result of DNA replication, and by a mitosis phase, in which the cell divides into two daughter cells. To simulate the growth phase, we consider that each *i*-cell has a total volume $V_{i}(t)$, whose temporal evolution is given by:

1 $$\begin{aligned} \dfrac{\text {d}V_{i}(t)}{\text {d}t} = \alpha _{i}(P_{in}(t),F_{c_{net_{i}}}(t))V_{i}(t), \end{aligned}$$

where $\alpha _{i}(P_{in}(t),F_{c_{\text {net}_{i}}}(t))$ is the growth rate of the *i*-cell, which we assume that depends on the luminal pressure ($P_\text {in}(t)$) and the net force exerted on the cell ($F_{c_{\text {net}_{i}}}(t)$). In this expression, cell growth is exponential and dependent on cell size according to experimental evidence that growth rates increase with cell size throughout the cell cycle [27, 32, 46].

Here, we assume the value of the growth rate is influenced by the luminal pressure $P_\text {in}(t)$ and by the net force exerted on the cell ($F_{c_{\text {net}_{i}}}(t)$). The variability of the growth rate during lumen morphogenesis has been experimentally observed in cell cultures in which Madin-Darby canine kidney cells (MDCK) [16] and primary pancreatic ductal epithelial cells (PDCs) [52] slowed their proliferation when they initiated the lumen. The onset of the lumen is determined with the initiation of the luminal pressure. Therefore, when there is no luminal pressure, the cell cycle time decreases so it can generate a closed volume to secrete fluid and form a lumen de novo. By contrast, when the luminal pressure increases, the cells do not need to divide as quickly as before, so the growth rate decreases. The growth rate $\alpha _{i}(P_\text {in}(t),F_{c_{\text {net}_{i}}}(t))$ is obtained from:

2 $$\begin{aligned} \alpha _{i}(P_\text {in}(t),F_{c_{\text {net}_{i}}}(t)) = {\left\{ \begin{array}{ll} 1 / T_\text {ini} (1+a_{i}(F_{c_{\text {net}_{i}}}(t))), P_\text {in}(t) = 0,\\ 1 / T_\text {pol} (1+a_{i}(F_{c_{\text {net}_{i}}}(t))), P_\text {in}(t)> 0, \end{array}\right. } \end{aligned}$$

where $P_{in}(t)$ is the luminal pressure and $T_{pol}$ and $T_\text {ini}$ are constants related to the cell cycle time ($T_\text {pol}>T_\text {ini}$). Second, we consider that the value of $\alpha _{i}(P_\text {in}(t),F_{c_{\text {net}_{i}}}(t))$ is influenced by $a_{i}(F_{c_{\text {net}_{i}}}(t))$ depending on the net cell force supported by the cell ($F_{c_{\text {net}_{i}}}(t)$). In this regard, the ECM stiffness regulates the magnitude of the net cell force, and an increase in cytoskeletal tension, mediated by sustained matrix stiffness, promotes growth [35]. Moreover, the compliance of the matrix acts as a cell-cycle inhibitor and matrix stiffening increases cell proliferation [28, 36, 53, 54] and cell cycle progression [25]. To model this mechanoregulation, we include a variation in the growth rate as a function of the net cell force as follows:

3 $$\begin{aligned} a_{i}(F_{c_{\text {net}_{i}}}(t)) = {\left\{ \begin{array}{ll} 0 , F_{c_{\text {net}_{i}}}(t) < F_\text {bottom},\\ \left( F_{c_{\text {net}_{i}}}(t) - F_\text {bottom}\right) \dfrac{a_\text {max}}{F_\text {top} - F_\text {bottom}} , F_\text {bottom} \le F_{c_{\text {net}_{i}}}(t) \le F_\text {top},\\ a_\text {max}, F_{c_{\text {net}_{i}}}(t) > F_\text {top}, \end{array}\right. } \end{aligned}$$

where $F_{c_{\text {net}_{i}}}(t)$ is the net cell force, $a_\text {max}$ is the maximum variation in the growth rate, and $F_\text {bottom}$ and $F_{top}$ are approximately the mean values of net cell forces when matrix density is low and high respectively.

Last, to account for biological variability, we let the daughter cell growth rate $\alpha _{i}(P_\text {in}(t),F_{c_{\text {net}_{i}}}(t))$ vary randomly between $[-20, 20]$% based on a normal probability distribution around the progenitor cell’s value.

When the volume of the cell reaches twice the value of its initial volume, DNA replication is concluded, and the cell divides. Spatially controlled division is a fundamental condition to maintain the lumen architecture and to enhance its growth by enlarging the lumen volume. In this regard, a complex molecularly controlled process regulates the spindle orientation, so mitosis occurs in the plane of the monolayer [22, 26, 31, 37, 41, 55]. Here, we distinguish division between nonpolarized cells and polarized cells. Nonpolarized cells are those that have not yet formed a lumen, and polarized cells are those that belong to a lumen and face it. In the case of nonpolarized cells, the division direction is chosen randomly. Polarized cell division is performed using a random cleavage plane that contains the line that passes through the cell center and the lumen center of mass. The position of the two daughter cells $(\mathbf {x}_\text {daughters})$ are calculated similarly to other models [21, 44] from the center of the parent cell $\mathbf {x}_\text {parent}$ at:

4 $$\begin{aligned} \mathbf {x}_\text {daughters} = \mathbf {x}_\text {parent} \pm \left( R_{c} - \dfrac{1}{\root 3 \of {2}}R_{c} \right) \mathbf {n}, \end{aligned}$$

where $R_{c}$ is the radius of the parent cell and $\mathbf {n}$ is the unit orientation vector. When a nonpolarized cell divides, the unit orientation vector $\mathbf {n}$ is chosen randomly. However, when a polarized cell divides, the unit orientation vector $\mathbf {n}$ is normal to the random cleavage plane that contains the line that passes through the cell center and the lumen center of mass.

After cell division, the cell decides whether to remain active and continue in the cell cycle to divide again or to become inactive within the cell cycle, namely restriction point [3]. In the model, at this point, either or both of the daughter cells can enter a quiescent state. Thus, we introduce a variable called $state_{i}$ to register for each cell whether they are active or inactive. The probability that a *i*-cell enters a quiescent state is

5 $$\begin{aligned} P(Q)_{i} = b\cdot c_{\text {steps}_{i}}, \end{aligned}$$

where *b* is a probability parameter and $c_{\text {steps}_{i}}$ is the number of times the cell has divided. Each time a cell divides, the value of $c_{\text {steps}_{i}}$ of its daughter cells increases by one. Thus, a random number in the interval [0,1] is generated for each daughter cell, and if it is lower than their probability $P(Q)_{i}$, the corresponding daughter cell enters into a quiescence state. When a cell becomes inactive, it implies that it does not grow ($\alpha _{i} = 0$) and, therefore, its growth rate does not follow equation 2. Otherwise, the cell continues in the cycle and starts growing to double its volume again and then divide.

### Fluid secretion

**Fig. 2:**
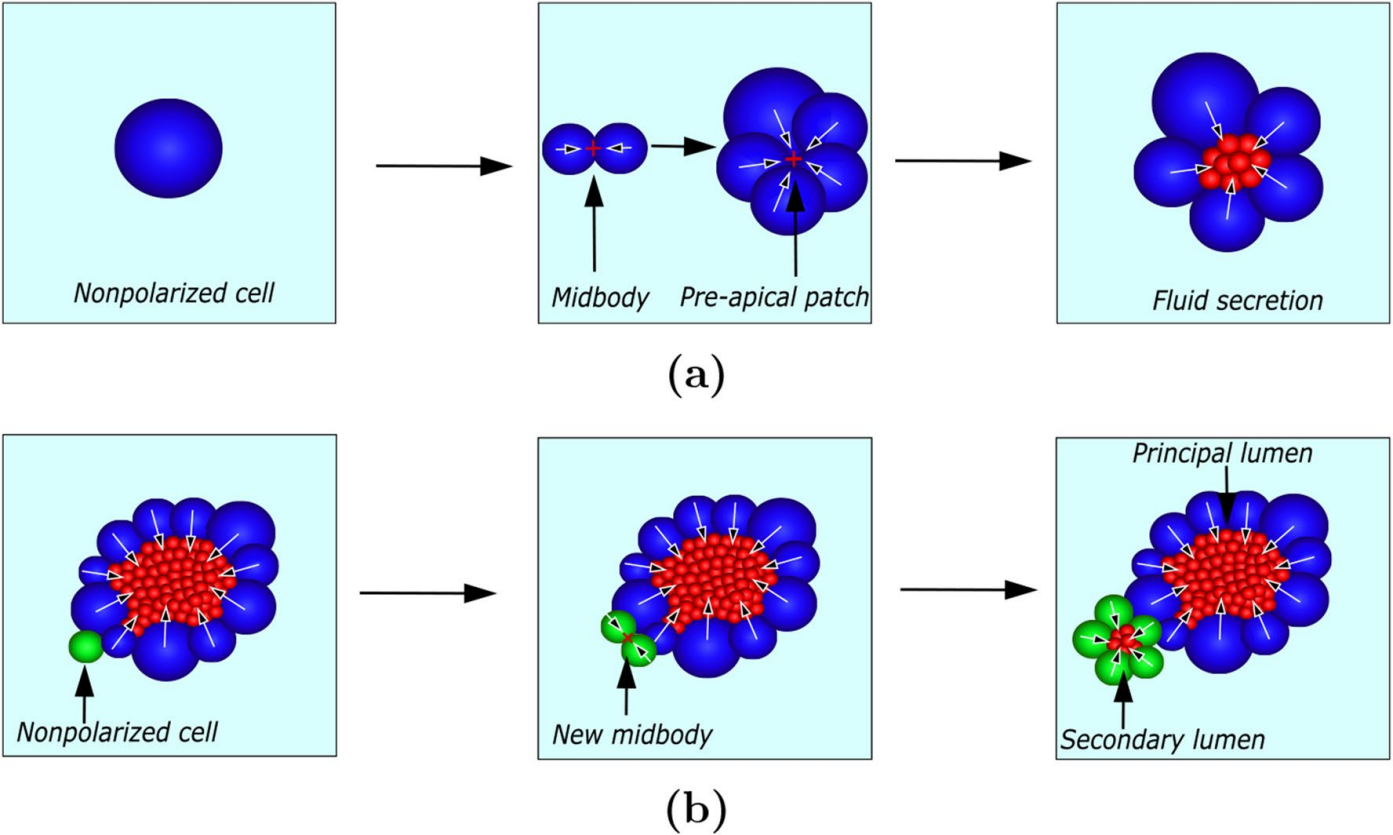
Sketch of the model for lumen morphogenesis. Case (**a**) shows how the lumen is formed beginning from an individual cell, and case (**b**) shows the formation of a secondary lumen. The arrows indicate the apical-basal polarization of cells

One of the key aspects of the model is how cells create the lumen. To generate a luminal domain de novo, neighboring cells must coordinate to secrete fluid into a common site, and that common site could be the midbody created during mitosis [11]. The midbody is a transient structure formed in the last phases of cell division to complete the separation between cells [24]. This landmark determines the apical-basal polarization of the cells and, therefore, the site where the cells will secrete to create the lumen. To contemplate the polarization of cells, we introduce a variable called $polarized_{i}$ that registers for each cell whether they are polarized or nonpolarized. Consider an initial active nonpolarized cell (Fig. 2a). When this cell divides, it generates a midbody, and the two daughter cells polarize with respect to that point. Then, the cells need to form a closed volume, the preapical patch (PAP) [5], to be able to generate hydrostatic pressure. We consider that the PAP is formed when the number of cells is equal to a specific value ($n_{t_{c}}$). With subsequent cell division, the number of cells increases, and the cells form the PAP. Once the initiation site for the lumen is created, the cells secrete fluid into that point to open the lumen. Each polarized cell, after a period of time of fluid production $\Delta t_\text {exo}$, secretes fluid into the lumen. To model the luminal fluid, cells generate a certain number of particles. First, when the lumen does not yet have any fluid, the cell secretes into the initiation site where the lumen will be generated. Then, when another cell secretes, some random particles are duplicated inside the lumen to recreate the increment in the fluid volume. The position of the new particles is obtained from the position of the particle that duplicates in a similar way to cell division. As a result of the cells’ secretion, the luminal hydrostatic pressure increases, and the lumen grows. The luminal hydrostatic pressure ($P_\text {in}$) is estimated by the mean of the particle’s net force ($F_\text {in}$). We hypothesize that there is a maximum luminal hydrostatic pressure above which cells cannot pump any more fluid into the lumen. Thus, cells can only secrete when the force generated by hydrostatic pressure, estimated through the mean particle net force, is below a threshold $F_\text {lim}$. This makes lumen formation a dynamic process of phases in which polarized cells can secrete, thereby increasing the hydrostatic, and phases in which polarized cells are not able to secrete due to the high pressure.

Cells remain polarized as long as they face either the midbody or the lumen. However, due to subsequent cell division or mechanical interactions, a cell can leave the lumen and no longer face it (green cell in Fig. 2b). This cell is now nonpolarized, and depending on whether it is active or not within the cell cycle, it may create a secondary lumen. If it is active, the process is equivalent to the previous case: a new midbody is established when the cell divides and the cells polarize with respect to that point, create a preapical patch, and secrete into the area to generate the lumen. In this case, when the initial nonpolarized cell polarizes to create a new lumen, the number of times that the cell has divided $c_{\text {steps}_{i}}$ is reset to prevent its daughter cells from entering quiescence and being unable to form the new lumen. On the other hand, if it is not active, the cell will not form a new lumen.

### Mechanical interactions

Mechanical interactions between agents make them move and change their positions. We illustrate how the cell position $\mathbf {x}_{c}$ and particle position $\mathbf {x}_{p}$ are calculated. Let $\mathbf {N}_{c}$ be the set of cells $\mathbf {N}_{c} = \{1, \ldots , N_{c}\}$, and let $\mathbf {N}_{p}$ be the set of particles $\mathbf {N}_{p} = \{1, \ldots , N_{p}\}$. First, the velocity of each *i*-cell $\mathbf {v}_{c_{i}}$ and each *k*-particle $\mathbf {v}_{p_{k}}$ are calculated from the balance of forces:

6 $$\begin{aligned} m_{c_{i}} \dfrac{\text {d}\mathbf {v}_{c_{i}}}{\text {d}t} = \sum _{j\in \mathbf {N}_{c}} \left( \mathbf {F}_{c_{i}c_{j}}\right) + \sum _{j\in \mathbf {N}_{p}}\left( \mathbf {F}_{c_{i}p_{j}}\right) + \mathbf {F}_{c_{i_{drag}}} \approx 0, \end{aligned}$$

7 $$\begin{aligned} m_{p_{k}} \dfrac{\text {d}\mathbf {v}_{p_{k}}}{dt} = \sum _{j\in \mathbf {N}_{p}} \left( \mathbf {F}_{p_{k}p_{j}}\right) + \sum _{j\in \mathbf {N}_{c}}\left( \mathbf {F}_{p_{k}c_{j}}\right) + \mathbf {F}_{p_{k_{drag}}} \approx 0. \end{aligned}$$

Here, $m_{c_{i}}$ and $m_{p_{k}}$ are the cell and particle masses, respectively, $\mathbf {F}_{c_{i}c_{j}}$ represents cell-cell interaction force, $\mathbf {F}_{c_{i}p_{j}}$ indicates the cell-particle interaction force, $\mathbf {F}_{p_{k}p_{j}}$ is the particle-particle interaction force, $\mathbf {F}_{p_{k}c_{j}}$ denotes the particle-cell interaction force and $\mathbf {F}_{c_{i_\text {drag}}}$ and $\mathbf {F}_{p _{k_\text {drag}}}$ are the friction of the cell and particle with the extracellular matrix, respectively. Random cues, such as chemotaxis, or random walk are neglected. Also, the inertial terms $m_{c_{i}} \dfrac{\text {d}\mathbf {v}_{c_{i}}}{\text {d}t}$ and $m_{p_{k}} \dfrac{\text {d}\mathbf {v}_{p_{k}}}{\text {d}t}$ are neglected because $Re<<1$. The drag forces $\mathbf {F}_{c_{i_\text {drag}}}$ and $\mathbf {F}_{p_{k_\text {drag}}}$ are obtained from Stoke’s law:

8 $$\begin{aligned} \mathbf {F}_{c_{i_\text {drag}}} = - 6\pi \eta R_{c_{i}} \mathbf {v}_{c_{i}}, \end{aligned}$$

9 $$\begin{aligned} \mathbf {F}_{p_{k_\text {drag}}} = - 6\pi \eta R_{p} \mathbf {v}_{p_{k}}, \end{aligned}$$

where $\eta$ is the dynamic viscosity of the extracellular matrix, $R_{c_{i}}$ is the radius of the *i*-cell, $R_{p}$ is the radius of the particle and $\mathbf {v}_{c_{i}}$ and $\mathbf {v}_{p_{k}}$ are the velocities of the *i*-cell and *k*-particle.

Cell-cell interaction forces are usually modeled as repulsive-attractive forces. The repulsion between cells arises from cell resistance to deformation when their membranes touch, and the attractive forces are the result of the junctions that cells form between themselves through specialized protein complexes [17]. In the case of particles, the repulsive-attractive forces represent the intermolecular forces in fluids. Accordingly, we modeled the interaction forces $\mathbf {F}_{\delta _{i} \gamma _{j}}$ (both subindexes $\delta$ and $\gamma$ denote *c* or *p*, depending on whether the *i* and *j* agents are cells (*c*) or particles (*p*)) following [34], as follows:

10 $$\begin{aligned} \mathbf {F}_{\delta _{i}\gamma _{j}} = F_{\delta \gamma } \dfrac{\mathbf {r}_{ij}}{||\mathbf {r}_{ij}||} \end{aligned}$$

where:

11 $$\begin{aligned} \mathbf {r}_{ij} = \mathbf {x}_{\gamma _{j}} - \mathbf {x}_{\delta _{i}}, \end{aligned}$$

and:

12 $$\begin{aligned} F_{\delta \gamma } = {\left\{ \begin{array}{ll} F_{\text {rep}_{\delta \gamma }} \chi (-s)^{3/2}, s<0\;{(\text {repulsion})},\\ - F_{\text {adh}_{\delta \gamma }} \chi \{(s+s_{0})e^{-\lambda (s+s_{0})^2} - v_{0}e^{-\lambda x^{2}}\}, s\ge 0 \;{(\text {adhesion})}. \end{array}\right. } \end{aligned}$$

Consequently, $\chi$, *s*, $x_{0}$ and $v_{0}$ are defined as:

13 $$\begin{aligned}&\chi = \dfrac{R_{\delta _{i}}}{2}\left( \dfrac{1}{R_{\delta _{i}}}+ \dfrac{1}{R_{\gamma _{j}}}\right) ,&s = \dfrac{d-min_\text {dist}}{R_{\delta _{i}}}, \end{aligned}$$

14 $$\begin{aligned}&x_{0} = \sqrt{\dfrac{1}{2\lambda }},&v_{0} = x_{0} e^{-\lambda x^{2}_{0}}. \end{aligned}$$

$F_{\text {rep}_{\delta \gamma }}$ and $F_{\text {adh}_{\delta \gamma }}$ are the strengths of the adhesive and repulsive forces, respectively. $\mathbf {r}_{ij}$ is the distance between the centers of the agents, and $R_{\delta _{i}}$ and $R_{\gamma _{j}}$ are the radii of the corresponding agents. $x_{0}$, $v_{0}$ and $\lambda$ are matching constants, and $\chi$ is a geometric correction factor. The value of $min_\text {dist} = -0.1 R_{\delta _{i}}$ is chosen such that the equilibrium state where the adhesive and repulsive forces are balanced is slightly less than zero, following [34], and $d = ||\mathbf {r}_{ij}|| - R_{\delta _{i}} -R_{\gamma _{j}}$ is the distance between the agents’ surfaces.

Since cells do not present any attraction towards the lumen fluid, the interaction force between cells and particles is only repulsive ($F_{\text {adh}_{cp}}=F_{\text {adh}_{pc}}=0$). Thus, the cell net forces $F_{c_{\text {net}_{i}}}(t)$ are computed from:

15 $$\begin{aligned} F_{c_{\text {net}_{i}}}(t) = |\sum _{j\in \mathbf {N}_{c}} \left( \mathbf {F}_{c_{i}c_{j}}\right) + \sum _{j\in \mathbf {N}_{p}}\left( \mathbf {F}_{c_{i}p_{j}}\right) |, \end{aligned}$$

and the mean interactive cells’ net force during the simulation as follows:

16 $$\begin{aligned} \bar{F}_{c} = mean\left( \dfrac{\sum _{j\in \mathbf {N}_{c}} \left( F_{c_{\text {net}_{i}}}(t)\right) }{n_{c}(t)}\right) , \end{aligned}$$

with $n_{c}(t)$ the number of cells.

Finally, the velocity of the *i*-cell and the *k*-particle at time *t* can be calculated explicitly:

17 $$\begin{aligned} \dfrac{d\mathbf {x}_{c_{i}}(t)}{dt} = \mathbf {v}_{c_{i}}(t) = \dfrac{1}{6\pi \eta R_{c_{i}}} \left( \sum _{j\in \mathbf {N}_{c}} \left( \mathbf {F}_{c_{i}c_{j}}\right) + \sum _{j\in \mathbf {N}_{p}}\left( \mathbf {F}_{c_{i}p_{j}}\right) \right) , \end{aligned}$$

18 $$\begin{aligned} \dfrac{d\mathbf {x}_{p_{k}}(t)}{dt} = \mathbf {v}_{p_{k}}(t) = \dfrac{1}{6\pi \eta R_{p}} \left( \sum _{j\in \mathbf {N}_{p}} \left( \mathbf {F}_{p_{k}p_{j}}\right) + \sum _{j\in \mathbf {N}_{c}}\left( \mathbf {F}_{p_{k}c_{j}}\right) \right) . \end{aligned}$$

### Implementation

**Fig. 3:**
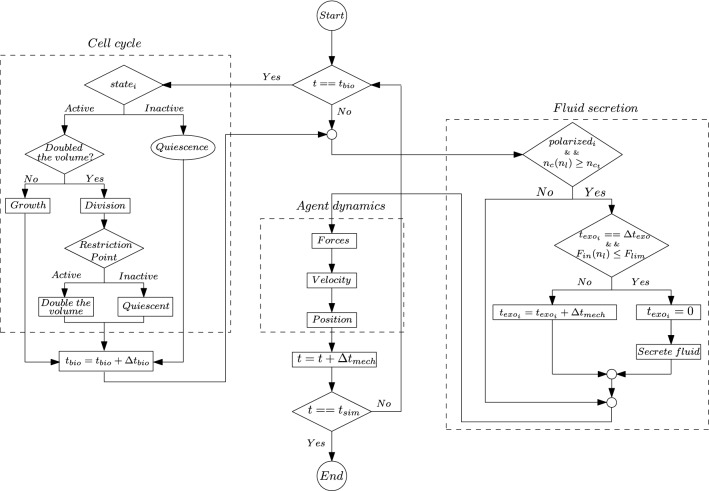
A simplified flowchart of the implemented algorithm. First, we check if the current simulated time *t* is equal to the time at which the cell cycle has to be solved $t_{bio}$. If that is the case, the cell cycle is solved for every cell. In the cell cycle, for each cell, we check whether it is active or inactive. If it is active, we evaluate if the cell has already doubled in volume and is ready to divide or if it has to continue growing. After division, the restriction point decides whether each daughter cell remains active and prepares to double in volume or enters into a quiescent state. If the cell is inactive, it remains quiescent. After the cell cycle has finished, $t_{bio}$ is increased. Then, we check if cells are polarized and if the number of cells in each lumen is higher than $n_{c_{t}}$. For each lumen which meet that condition, we check for each cell polarized around that lumen if it is its time to secrete fluid and if the lumen pressure is lower than $F_\text {lim}$. If the cell secretes, we reset its time of fluid production $t_{\text {exo}_{i}}$ and secrete fluid. If not, the cell produces fluid by increasing $t_{\text {exo}_{i}}$. Afterward, we solve the agent dynamics. We obtain the forces produced by mechanical interactions and the velocity of the agents and update the agents’ positions. Finally, we increase the simulated time; if the stipulated simulation time $t_\text {sim}$ is reached, the simulation is complete; otherwise, we repeat the algorithm

Mechanical interactions occur faster than biological processes ($\Delta t_\text {mech} < \Delta t_\text {bio}$), which allows them to be uncoupled and implemented with different time steps [6, 21]. The fluid secretion and agent dynamics are solved every $\Delta t_\text {mech}=0.01\;min$, and when the current simulated time (*t*) increases $\Delta t_\text {bio} = 6\;min$ ($t=t_\text {bio}$), the cell cycle is solved for each cell along with the fluid secretion and the agent dynamics. Figure 3 presents a simplified flowchart of the implemented algorithm. Initially, we begin the simulations with an active cell. The variable $state_{i}$ stores for each cell whether they are active or inactive within the cell cycle. After division, in the restriction point, cells can reenter in the cell cycle or become inactive, so the variable $state_{i}$ is updated for each daughter cell. If the cell becomes inactive, it enters into a quiescent phase and does not grow anymore ($\alpha _{i}=0$). In the fluid secretion part, the variable $polarized_{i}$ accounts whether cells are polarized or nonpolarized, and $n_{l}$ refers to the number of lumens in the organoid, therefore, $n_{c}(n_{l})$ is the number of cells in the $n_{l}$ lumen. Moreover, we track the fluid production time $t_{exo_{i}}$ for each polarized cell and the luminal hydrostatic pressure $F_\text {in}(n_{l})$ in the $n_{l}$ lumen. If the cell secretes fluid, we reset its fluid production time $t_{exo_{i}}$. Finally, after fluid secretion, we solve the agent dynamics.

To evaluate the predictive capacity of the model, we replicate the experiment developed by [35], who studied the influence of matrix rigidity in the lumen formed by MCF10A cells and concluded that matrix stiffening compromises tissue organization, inhibits lumen formation and enhances growth. However, there was no clear conclusion about the intrinsic mechanisms that regulate this process. Here, we focus on reproducing the results by comparing the lumen formation in a low-density matrix and a high-density matrix. Different techniques have been employed to model the extracellular matrix (e.g., the smoothed particle hydrodynamics (SPH) [23]). Here. we model the ECM through a uniform dynamic viscosity and relate the density of the ECM with the dynamic viscosity based on [47], which presents a characterization of crosslinked collagen-based hydrogels. The low-density matrix represents the 4 mg/ml collagen concentration, with a dynamic viscosity of approximately 20 Pa s ($\eta _\text {low}$) [47]. Regarding the high-density matrix, we consider a dynamic viscosity of 100 Pa s ($\eta _\text {high}$), which corresponds to a five-fold increase in the dynamic viscosity with respect to the low-density matrix. Moreover, to analyse the trend of the lumen evolution under different-density matrices, we include an intermediate-density matrix with a dynamic viscosity of 50 Pa s for comparative purposes.

Finally, we initiate the simulations with one cell of radius 10 $\mu m$, then create a random seed and run the simulation for 7 days. Thus, we first performed 20 simulations with the low-density matrix and then, with the random seeds generated for each, we executed the equivalent simulations for the intermediate- and high-density matrices. The agent surfaces were discretized and then processed with alpha shapes [15]. The parameters used for the simulations are shown in Table 1. The code was fully implemented in Matlab R2019a.

**Table 1:** Parameters of the model

Parameter	Description	Value	Source
$\Delta t_\text {exo}$	Fluid production time	5 h	Estimated
$\eta _\text {low}$	Dynamic viscosity of the low-density matrix	20 Pa s	[47]
$\eta _\text {high}$	Dynamic viscosity of the high-density matrix	100 Pa s	[35, 47], estimated
$F_\text {bottom}$	Mean value of the cells’ net force in the low-density matrix	0.42 pN	Estimated
$F_\text {top}$	Mean value of the cells’ net force in the high-density matrix	1.17 pN	Estimated
$T_\text {ini}$	Cell cycle time constant	30 h	Estimated
$T_\text {pol}$	Cell cycle time constant	100 h	Estimated
$n_{t_{c}}$	Number of cells to form the PAP	5	Estimated
$a_\text {max}$	Maximum variation in the growth rate	60%	[35], estimated
*b*	Quiescence probability	0.1	Estimated
$R_{p}$	Particle radius	2 $\mu m$	Estimated
$F_{\text {rep}_{cc}}$	Cell-cell repulsive force	– 4.80 pN	[34], estimated
$F_{\text {adh}_{cc}}$	Cell-cell adhesive force	24 pN	[34], estimated
$F_{\text {rep}_{pp}}$	Particle-particle repulsive force	– 4.80 pN	[34], estimated
$F_{\text {adh}_{pp}}$	Particle-particle adhesive force	24 pN	[34], estimated
$F_{\text {rep}_{cp}}$	Cell-particle repulsive force	0.60 pN	[34], estimated
$F_{\text {adh}_{cp}}$	Cell-particle adhesive force	0	[34], estimated
$\lambda$	Matching constant of the potential function	7	[34]
$F_\text {lim}$	Force threshold for cell secretion	0.07 pN	Estimated

## Results

### Lumen morphogenesis requires a low-density matrix to be accomplished

**Fig. 4:**
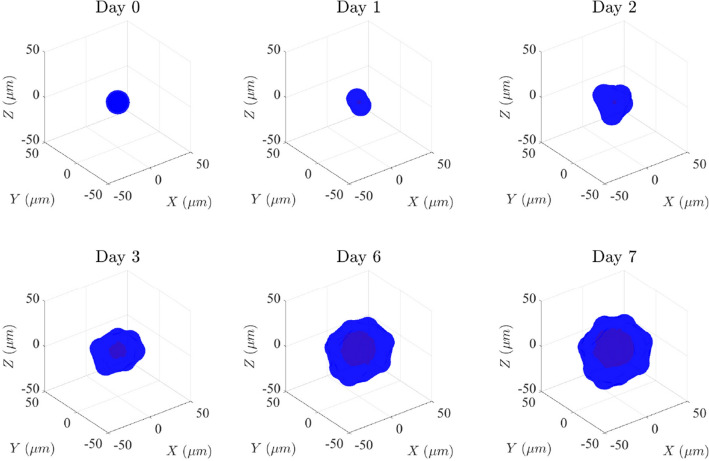
Evolution of a simulated cyst in the low-density matrix ($\eta = 20\; Pas$) during 7 days. Cells are represented in blue and the lumen fluid in red (color figure online)

First, we studied lumen formation in the low-density matrix with a dynamic viscosity of 20 Pa s ($\eta _\text {low}$) (S1 Video). We simulated the evolution of one lumen during 7 days (Fig. 4). The simulation began with only one cell, and on the first day, it grew and then divided into two cells (day 1). At this moment, a midbody was created (red dot), which determined the apical-basal polarization of the cells and the common site where cells would secrete. At day 2, the cells divided around the midbody to create a closed volume. Then, cells began to secrete and create a small lumen. At day 3, the lumen was formed by eight cells and was already in a state of hydrostatic pressure. Between day 3 and day 6 the lumen continued growing, increasing its volume by a factor of 9.7 times on day 6 compared to day 3, and the number of cells increased to 16. Finally, at day 7, a cyst with a single lumen formed by a monolayer of 16 cells was achieved.

### Increasing the matrix density hinders lumen morphogenesis and produces an aberrant multiluminal architecture

**Fig. 5:**
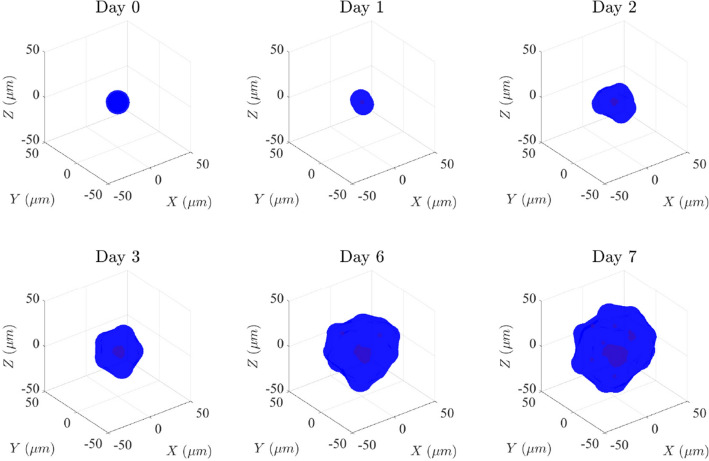
Evolution of a simulated cyst in the high-density matrix ($\eta = 100\; Pas$). Cells are represented in blue and the lumen fluid in red (color figure online)

Then, we investigated, with the same seed of random numbers, the evolution of the lumen during 7 days of simulation in a dense matrix with a dynamic viscosity of 100 Pas ($\eta _\text {high}$) (Fig. 5 and S2 Video). Again, the simulation began with one cell at day 0, which grew and divided. At day 1, the midbody was already generated. At day 2, due to the fact that in the high-density matrix the cells’ net forces are higher than those in the low-density matrix, the growth rate of cells had increased, so the number of cells increased to 8. At this point, a closed volume for lumen initiation was created, and cells secreted fluid into the lumen, generating a small lumen. In the next 24 hours, cells continued secreting, and the lumen volume increased slightly. Between day 3 and day 6, significant changes were observed. The number of cells increased from 8 to 24. However, the lumen volume increased by only a factor of 3 (the increment in the low-density matrix was approximately 9.7). The high-density matrix opposes cell movement, so the luminal hydrostatic pressure cannot displace the cells. Consequently, the pressure increases with successive secretions without enlarging the lumen, at times reaching the hydrostatic pressure limit at which cells cannot pump more fluid into the lumen. Moreover, as the lumen is small, cell divisions caused some cells to move into a second layer where they ceased to face the lumen. This change made them polarize to create two midbodies and later two new lumens. Finally, at day 7, a dysfunctional structure composed of 7 lumens and 53 cells was observed.

### Matrix density reduces the lumen volume

**Fig. 6:**
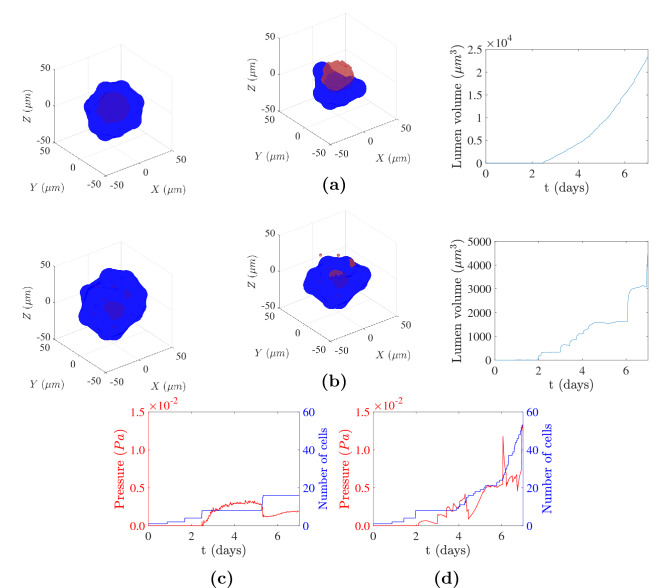
Cyst formed after 7 days of simulation in the low-density matrix in (**a**), and in the high-density matrix in (**b**). Full view of the cyst (left), a perspective of the cyst with only the cells positioned approximately below the center of the organoid (middle), and the evolution of the lumen volume (right). Cells are represented in blue and the lumen fluid in red. Evolution of the pressure and number of cells in the low-density matrix in (**c**) and in the high-density matrix in (**d**) (color figure online)

To compare the lumen formed in the low and high-density matrices, we show the structure achieved (Fig. 6a, b (left)) and a view of the lumen with only the cells that are situated approximately below the center of the organoid in both matrices (Fig. 6a, b (middle)) after 7 days of lumen formation. In the case of the low-density matrix, a cyst with a single regular lumen and 16 cells was developed. However, in the high-density matrix, 7 lumens were developed. The volume of the largest lumen for the high-density matrix was 80.64% smaller than that in the case of the low-density matrix, and the secondary lumen volume formed in the high-density matrix was 94.2% less than that of the principal lumen of the cyst. Moreover, non-linear increase of the lumen volume was obtained in the case of the low-density matrix (Fig. 6a (right)) since there were more cells to secrete fluid as the simulation time increased due to cell proliferation. In contrast, in the high-density matrix, the principal lumen volume grew discontinuously (Fig. 6b (right)), with higher increases in lumen volume as the simulation proceeded. The evolution of the pressure and the number of cells is represented in the low- and high-density matrix (Fig. 6c, d). The pressure was obtained integrating the interacting forces between the cells and the lumen fluid ($\mathbf {F}_\text {cp}$) divided by the lumen surface. The pressure increased after day two with successive cell secretions. Thus, the pressure in the low-density matrix (Fig. 6c) increased when cell secreted fluid and then decreased as a consequence of lumen expansion. After day 5, a significant decrease in pressure was developed since cells proliferated and generated more inner volume. In contrast, in the high-density matrix (Fig. 6d), with each cell secretion the pressure increased more sharply. In this situation, little lumen expansion was produced and, therefore, the pressure increased with time. In this case, the total population of cells increased 3.3 times compared to the low-density matrix, from 16 cells to 53 cells.

**Fig. 7:**
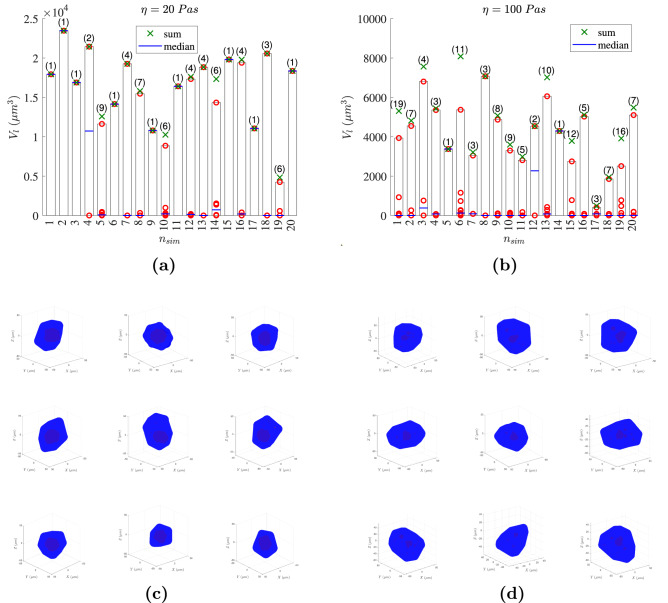
Volume of the lumen after 7 days for each of the 20 simulations in the low-density matrix in (**a**) and in the high-density matrix in (**b**). The numbers in parentheses indicate the number of lumens produced, the red circles represent the discrete volume of each lumen, the green crosses show the sum of the volumes of all lumens in each simulation and the blue line is the median. The bars represent the volume of the largest lumen, which are plotted for the sake of the visualization (note the difference between the scales of the graphs). A representation of the cyst formed in simulations 1, 2, 3, 4, 7, 13, 15, 17, and 20 is shown in the case of the low-density matrix in (**c**) and in the case of the high-density matrix in (**d**) (color figure online)

To analyze the robustness of the model, we show (Fig. 7) the lumen volume after 7 days for the 20 simulations performed in the low and high-density matrices^1^ (see S3 Figure for a representation of the lumen volume after 7 days for each simulation performed in an intermediate-density matrix ($\eta =50$ Pas)). In the case of the low-density matrix (Fig. 7a), 9 of 20 simulations formed a single large lumen, and 11 simulations formed a principal large lumen with some additional small ones. The median value of the number of lumens was 2.5. When the sum of the lumens coincides with the higher lumen volume value, the volumes of the secondary lumens are insignificant compared to that of the principal lumen. In the case of the high-density matrix (Fig. 7b), only two cases produced a single lumen, and the median number of lumens increased to 6. In all simulations in the high-density matrix, the higher lumen volume is significantly smaller than that in the previous cases. Moreover, two main cases are identified, those with a small principal lumen with various lumens of negligible volume and those with a small principal lumen, some secondary lumens smaller than the principal but still significant and some minor lumens (for instance, simulations 1, 3, 6, 13, 15 and 19).

**Fig. 8:**
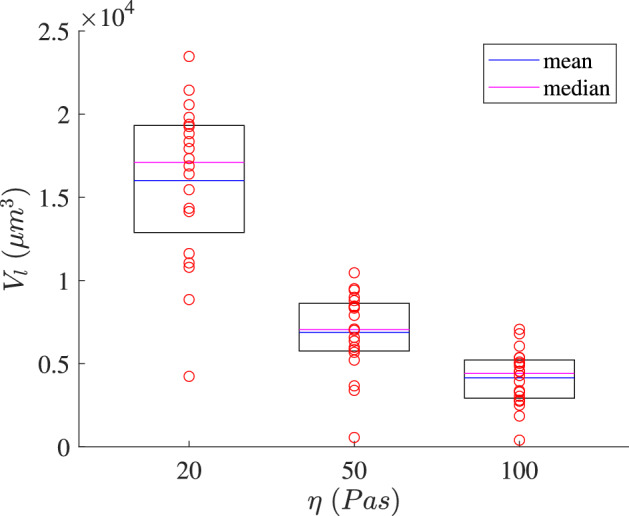
Higher lumen volume of the organoid after 7 days for each simulation in the low-, intermediate- and high-density matrices. Red circles are the discrete values of each simulation. In cases in which multiple lumens were produced, the red circle corresponds to the volume of the largest lumen. The top line of the box represents the 75th percentile and the bottom line the 25th percentile. The magenta line shows the mean and the blue line the median (color figure online)

We compare the higher lumen volume of the organoid obtained in each simulation after 7 days in the low-, intermediate- and high-density matrices for the 20 simulations performed (Fig. 8). The lumen volume obtained in the simulations in the low-density matrix presents a higher variability than that in the high-density matrix. Thus, the standard deviation is 2.8 times higher than that in the high-density matrix. However, the lumen volume achieved in most cases in the low-density matrix is significantly greater than that in the high-density matrix. Additionally, the median lumen volume difference between the low-density and high-density matrix is approximately 74.2%, between the low-density and intermediate-density matrix is approximately 57.0%, and 39.7% between the intermediate-density and high-density matrix.

**Fig. 9:**
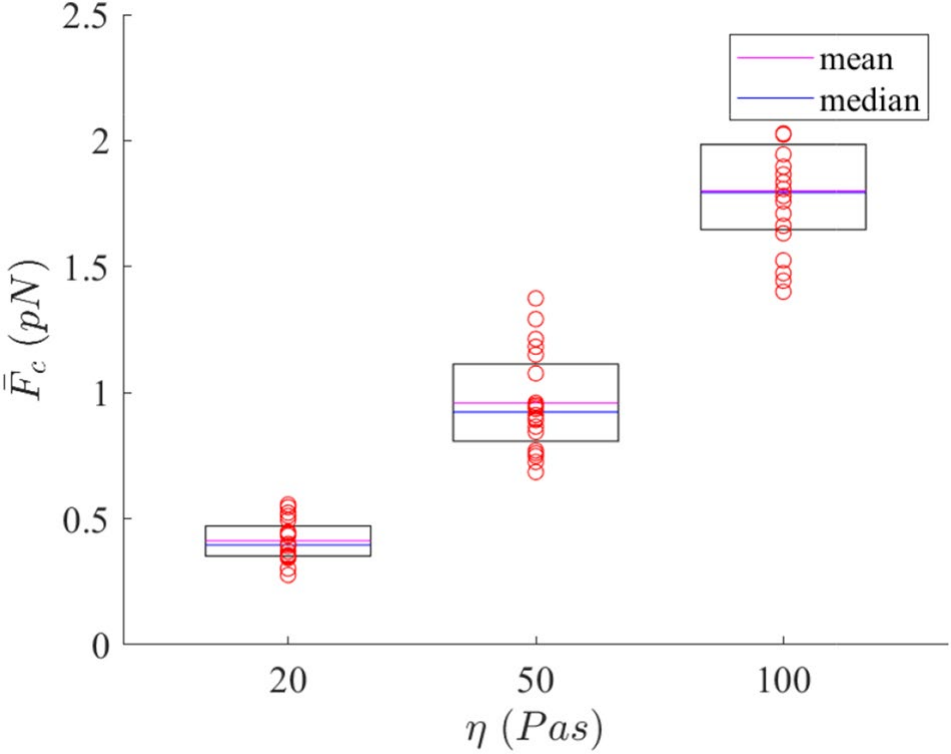
Mean value of the cells’ net forces for each simulation in the low-, intermediate- and high-density matrices. Red circles are the discrete values of each simulation. The top line of the box represents the 75th percentile and the bottom line the 25th percentile. The magenta line shows the mean value and the blue line the median (color figure online)

Finally, the mean interactive cells’ net force during the 7 days simulated for each simulation in the case of the low-, intermediate- and high-density matrices is presented in Fig. 9. The forces acting on cells are higher in the high-density matrix than in the low-density matrix. In particular, the median in the high-density matrix is approximately 4.5 times greater than that in the low-density matrix. Moreover, the standard deviation in the high-density matrix is 2.98 times higher than that in the low-density matrix. In the case of the intermediate-density matrix, the median is 2.3 times greater than in the low-density matrix but 0.51 times lower than in the high-density matrix.

## Discussion

We present a three-dimensional multi-agent-based model for lumen morphogenesis that introduces the regulatory role of the luminal hydrostatic pressure generated by the cells’ fluid secretion and the interaction with the extracellular matrix. These biophysical effects allow simulation of lumen morphogenesis under three matrices with different densities and determination of how the density influences its formation. We show that increasing the density of the matrix hinders lumen morphogenesis, increases the number of cells, and produces a dysfunctional architecture. Moreover, the model predicts normal lumen morphogenesis and aberrant multilumen formation related to tumor formation as a result of the increased density of the matrix.

In a high-density matrix, we find alteration of the lumen formation process that produces an aberrant structure. The main contribution to the disruption of normal lumen morphogenesis is the balance between the matrix and the forces derived from the luminal hydrostatic pressure. When the matrix density is high, the luminal hydrostatic pressure generated by the cell secretion cannot displace the cells as easily as when the matrix density is low since the matrix opposes to the cells’ movement. Consequently, the lumen remains diminished, and the hydrostatic pressure continues increasing with the successive cell secretion to a value at which point cells cannot pump more fluid into the lumen. In this situation, cell division does not contribute to creating luminal space because the lumen size is small, and its surface is fully covered by overlapped cells. Conversely, as there is no space around the lumen surface, cell division forces some cells to leave the monolayer. At this point, the cells that do not face the lumen attempt to polarize to create a new lumen. Finally, a dysfunctional structure with numerous small lumens is formed. This aberrant architecture is associated with tumor pathogenesis and is found in many carcinomas [12]. Therefore, we conclude that the relationship between the matrix density and the luminal hydrostatic pressure is crucial and might help to determine the development of tumor pathogenesis [35].

Moreover, the matrix density not only acts as a damping and determines the timescale of the problem, but also provides important mechanobiological feedback to cells. From a mechanobiological perspective, the second aspect that contributes to lumen malformation is the cell net force. We showed that cells’ net forces increase with matrix density (Fig. 9), and this increment in the net forces transforms the cells towards a malignant phenotype, which enhances the cell growth rate. The interplay between mechanical forces, extracellular matrix, and growth factors controls the cell cycle progression [25]. Thus, the compliance of the matrix acts as a cell-cycle inhibitor and matrix stiffening increases cell proliferation [28, 36, 53, 54]. Our model includes this effect and when the matrix density is higher, the cell net forces are higher and it makes cells increase their growth rate, therefore, the total number of cells increases with matrix density. This contribute to some cells to escape from the lumen they are facing and attempt to form a new lumen. Moreover, we showed that there is low variability in mean cell net force; therefore, it is strongly influenced by matrix density. Therefore, the density of the matrix acts not only as a relaxation time of the system, but also as a regulator of cells’ net forces, luminal pressure, morphology, and the formation of multiple lumens.

Our work replicates the experimental observations of [35]. They found that matrix rigidity compromises tissue organization, inhibits lumen formation, increases colony size, and increases cell forces. These four tendencies are observed in our results; in the case of the high-density matrix, an aberrant architecture with multiple lumens was obtained, the lumen volume was significantly smaller than that in the low-density matrix, and the number of cells and the cells’ forces increased.

In the proposed model, several simplifications have been considered. Cells are assumed to be nondeformable spheres; therefore, cell shape was not represented accurately. In a deformable model, we would have cell deformation around the lumen. However, apart from the shape representation, little differences in cells’ position are expected. This simplification reduces the computational cost of the simulations, but it does not affect the process of lumen morphogenesis. Here, we focused on simulating lumen morphogenesis from the beginning with an individual cell; therefore, we are focused on determining the position of each cell to understand their coordination rather than obtaining the exact representation of the cell shape. Moreover, we represented the lumen fluid through a particle-based simulation. With this approach, the mechanical properties of the fluid are not accurately described, specially concerning the rheological behavior of the lumen. Although this representation is a simplification, it allowed us to simulate the interaction between cells and the fluid in a developmental process in which the interface between cells and the fluid changes with time. Also, our model permits to simulate the formation of lumens de novo and the increase of the luminal fluid.

In our model, we characterized the extracellular matrix by means of the dynamic viscosity for the whole domain. With this approximation, we are considering the viscous properties of the matrix but not the elastic ones. Despite this simplification, our model allows us to study how the biophysical properties of the ECM, represented through its viscosity, affects the lumen size and morphology in a three-dimensional simulation. Both cells and particles experiment a drag force that opposes to their relative motion with the matrix. It may be reasonable to expect variation in the dynamic viscosity between the lumen and the outside of the cyst. A lower dynamic viscosity inside the lumen implies lower resistance to the motion of particles, and we might expect variation in the growth and size of the lumen. However, this aspect is already considered through the parameter representing the strength of the repulsive and adhesive forces in the particle-particle and particle-cell interactions. Thus, a reduction in dynamic viscosity in the lumen facilitates particle movement, which is equivalent to an increase in the magnitude of these interacting forces.

In this study we suggest that there is a maximum luminal pressure above which the cells cannot secrete. On one hand, an increase in the force threshold for cell secretion allows higher hydrostatic pressure, and the lumen might open. On the other hand, if the threshold is excessively restricted, cells cannot secrete, and the lumen remains diminished. Therefore, this parameter is set such that it allows the lumen to grow without opening.

This computational model is conceptualized as a tool for simulating lumen morphogenesis by cells in different organs. Thus, the parameters used in the model could be experimentally quantified and, therefore, enable adjustment of the model to simulate lumen morphogenesis in different scenarios. Specifically, the cell cycle constants of the model can be experimentally estimated to characterize diverse cell types that form a lumen. The fluid production time can be estimated by monitoring the volume of the lumen in cell cultures such that the evolution of the lumen volume obtained can be fitted with the experimental results.

## Conclusions

This work finds a strong correlation between the density of the extracellular matrix and lumen morphogenesis. Thus, an optimal density that provides adequate biomechanical conditions to form the lumen and to reach a proper structure may exist. We showed that cells require a low-density matrix to form a normal lumen. In this case, cell division and cell fluid secretion act in a coordinated manner to form a normal lumen. However, an increase in matrix density disrupts this coordination and promotes an aberrant multiluminal architecture. Therefore, matrix density provides crucial properties to regulate the deviation from normal lumen morphogenesis to tumorigenesis. Finally, we would like to highlight that this computer-based model has allowed to investigate different-mechanical scenarios representative of a relevant biological process as lumen morphogenesis, clearly demonstrating the possibilities of computational simulations in biological engineering reseach.

## Supplementary Information

Below is the link to the electronic supplementary material.

Supplementary file1 (AVI 14120 kb)

Supplementary file2 (AVI 14148 kb)

Supplementary file3 (EPS 129 kb)

## Footnotes

1 The cases of lumen morphogenesis in the low and high-density matrices presented previously correspond to the second simulation ($n_\text {sim} = 2$) in Fig. 7a, b respectively.
